# Knowledge and perceptions of religious leaders toward HIV prevention among young people in a resource-limited setting: A qualitative study

**DOI:** 10.21203/rs.3.rs-3442966/v1

**Published:** 2024-05-08

**Authors:** Tom Murungi, Irene Kunihira, Pamela Oyella, Moses Mugerwa, Peruth Gift, Mercy Jane Aceng, Lydia Abolo, Sean Steven Puleh

**Keywords:** Knowledge, Perceptions, Religious leaders, HIV prevention, Young people

## Abstract

**Background:**

Currently, 410,000 new HIV infections among youth occur worldwide, which is a significant public health issue. Members of the clergy can be trustworthy allies in the reduction of HIV infections among the youth. However, little is known regarding their knowledge as well as the perceptions they hold towards HIV prevention among young people. Thus, we explored the knowledge and perceptions of religious leaders regarding HIV prevention among young people (15–24 years) in Lira district.

**Methods:**

This was a cross-sectional qualitative study conducted among 20 religious leaders in March 2021 in Lira district. Religious leaders were sampled purposively and recruited from modern religions (beliefs influenced by Christianity or Islam) in Lira district. Guides for key informant interviews were utilized to gather information. Each interview was audio recorded, transcribed, and entered into NVivo version 12 software, and the data was then ready for analysis. The main themes were determined using thematic analysis.

**Results:**

Although a few individuals had some misconceptions, the majority of participants had good knowledge about the transmission and prevention of HIV. Participants knew awareness creation, abstinence, and faithfulness in marriage as HIV prevention strategies and held positive perceptions. Perceived barriers to HIV prevention involvement were lack of knowledge and training, and inadequate resources whereas motivating factors were; being respected, and trusted, and having easy access to young people.

**Conclusion:**

In conclusion, religious leaders show limited HIV prevention knowledge due to religious beliefs, but understand the importance of measures like abstinence. Despite challenges, their involvement is crucial. Addressing knowledge gaps and providing support is vital. Future efforts should emphasize both behavioral measures and interventions like condom use, Post Exposure Prophylaxis, and Pre-Exposure Prophylaxis.

## Introduction

The potential role of religious leaders and institutions in the HIV response remains a focal point of interest for policymakers and health professionals, given their historical significance in shaping HIV prevention initiatives in Africa during the late 20th century. In Uganda, spiritual leaders have historically played a significant role in HIV prevention efforts, particularly before the introduction of condoms and antiretroviral drugs, emphasizing behavioral approaches (2, 4). Recent studies have underscored the substantial contributions and reliability of spiritual leaders as partners in HIV prevention, particularly among young people, who constitute a significant proportion of new HIV infections globally (5–7).

Uganda boasts a substantial population affiliated with religious groups, affording religious leaders broader access to young people and a unique platform to promote moral teachings and behavior change. Despite growing evidence suggesting the influential role of religious leaders in HIV prevention among young people, there are still gaps in our understanding of their perceptions and knowledge regarding HIV prevention strategies.

Globally, HIV remains a significant public health challenge, with young people and adolescents bearing a disproportionate burden of new infections. In sub-Saharan Africa, including Uganda, HIV continues to contribute significantly to mortality and morbidity rates among young populations (9–12).

While previous research indicates that religious leaders possess relatively good knowledge of HIV prevention, there is evidence of persistent misconceptions and negative attitudes toward certain prevention methods among some religious leaders (13, 14). Moreover, studies have revealed a tendency among religious leaders to emphasize abstinence and faithfulness while providing limited support for condom use, despite the effectiveness of multi-strategy approaches to HIV prevention (7, 15).

Recognizing the pivotal role of religious leaders in the design and implementation of HIV prevention strategies, the Uganda AIDS Commission has urged their participation in the multisectoral response against HIV, aligning with global health sector strategies aimed at empowering communities and civil society to achieve HIV targets by 2030 (9, 16).

In light of the ongoing misconceptions and negative perceptions held by some religious leaders regarding HIV prevention, particularly in regions like Lira district where the religious sector has significantly contributed to reducing HIV infections among youth, there remains a critical gap in understanding the knowledge and perceptions of leaders from various religions regarding HIV prevention. This paper seeks to address this gap by providing insights into how the knowledge and perceptions of religious leaders can impact their role in HIV prevention and offering suggestions for strengthening their contributions to effective prevention strategies, particularly among young people. Thus, exploring the understanding and perceptions of religious leaders on HIV prevention represents a novel approach to identifying opportunities for enhancing prevention efforts.

## Methods and Materials

### Study Design and Setting

This was a cross-sectional qualitative study carried out in Lira district including Lira City in Northern Uganda in March 2021. The majority populations in Lira city and district are Christians and Muslims. A number of faith-based organizations support HIV prevention efforts in the area; most of them have their primary offices in Lira City (17). In Lira, the majority of people identify as Christians (more than 80%), with the rest population being either Pagan or Muslim (18). Lira district has an HIV prevalence of 7.2% among people aged 15–49 years (19). With the help of religious institutions, the government of Lira district has launched HIV prevention initiatives, including peer education, volunteer counselling and testing, and training for educators and other stakeholders (20, 21).

### Study Population

The study recruited religious leaders in Lira district, Northern Uganda. In this study, religious leaders were individuals who were formally ordained or appointed by religious authorities to guide, teach, or provide spiritual guidance to members of their religious community. Only religious leaders who belonged to the predominant religious denominations, such as Catholics, Anglicans, Pentecostals, and Muslims were included. They included pastors, bishops, sheikhs, imams, reverends and catechists.

### Inclusion and Exclusion Criteria

The study included religious leaders who belonged to the two most common religious affiliations (Christians and Muslims), held official posts, performed administrative tasks, and engaged in daily activities inside their institutions. The study did not include people who were ill and unable to speak or religious leaders who were busy conducting their pastoral services, such as fellowships and holding prayers at worship places. In addition, religious leaders who couldn’t participate in the study because of sickness or other reasons were excluded.

### Sampling Procedure and Sample Size

Religious leaders were purposefully sampled using maximum variation (22) because they were thought to know more due to their role within the religious organizations. Participants were approached face to face and invited to take part in the study, with an emphasis on ensuring representation from the two strata of the district (rural) and the city (urban), major religious denominations, and diverse leadership positions. The recruitment process continued until data saturation was reached, meaning that no new themes or insights were emerging from additional interviews, indicating that a comprehensive range of perspectives had been captured. Through the systematic application of these sampling criteria, the study aimed to obtain a rich and diverse dataset that reflected the varied perspectives of religious leaders on HIV prevention among young people within the community. Twenty (20) religious leaders were included in this study.

### Data Collection Tool and Procedure

The key informant interview guide was developed by the researchers following an extensive literature review. The tool had open-ended questions about the knowledge, concerns, and opinions held by various faith leaders on HIV prevention among youth aged 15–24 years. Ethical approval was obtained from the Gulu University Research Ethics Committee (GUREC-2021–34) whereas study site clearance was obtained from the District Health Officer (DHO) and the Resident District Commissioner (RDC) of Lira. Data were collected by four experienced research assistants who were closely supervised by the principal investigator. The research assistants were trained on ethical conduct, study tools, and data collection methods before data collection. While in the field, twenty religious leaders were purposively sampled, and consent for participation was obtained from each participant. Each interview was conducted by two research assistants. To guarantee the accuracy of the data, interviews were audio-recorded and notes were gathered. At all times, participant confidentiality was upheld. The interviews lasted about 45–60 minutes. During the interviews, both verbal and non-verbal clues were followed, and probing questions were asked to exhaust any possible responses from the participants.

Shown in Table 1 is the data collection tool used to conduct key informant interviews among religious leaders in Lira district.

### Data Management and Analysis

After the interviews, the researchers cross-checked for any unanswered questions and probed further for those that seemed unclear. All interviews and discussions were recorded, and notes were taken. The audio recordings were stored and protected by the principal investigator. For those done in the native tongue, each audio recording was translated into English after being verbatim transcribed. All data was entered into NVivo version 12 software, ready for onward analysis.

Each researcher employed thematic analysis to examine the data per the seven processes of data analysis, which include 1) transcription, 2) reading, and familiarization. Coding, theme-finding, theme-review, theme-naming, and concluding the analysis and result interpretation are the next steps (23). Two researchers independently read and immersed themselves in the qualitative data. They both took notes and highlighted important themes, patterns, or ideas that emerged during this initial familiarization process. The study team collaboratively developed a coding framework that captured the key themes in the data, which served as a guide for coding and organizing the data. The study team divided the data into manageable segments and assigned them to each researcher. Each individual coded the data according to the agreed-upon coding framework. Once each researcher had completed the initial coding, the study team looked for areas of agreement and divergence in the coding. Where discrepancies arose, the team engaged in discussions to understand each other’s perspectives and reached a consensus on the most appropriate codes. Based on the discussions, we refined and revised the coding framework. After achieving consensus on the coding framework, the team analyzed the data mutually to identify recurring themes and explore the underlying meaning of the data.

#### Ethical Consideration

The research was carried out following the guidelines of Gulu University Research and Ethics Committee that approved the study. Site clearance was obtained from the District Health Officer and further consent was sought from the district headquarters of various religious denominations. All participants received written informed consent forms outlining the study’s terms and conditions, which they were expected to comprehend and decide whether to accept or decline before signing. In this study, the interview guides did not include any names or other identifying information. Additionally, each participant underwent a private, silent interview, and all soft copies of the data were kept on password-protected computers and hard drives to prevent unauthorized access.

## Results

### Socio-demographic characteristics of the participants.

In Table 2, the socio-demographic characteristics of the respondents are presented quantitatively. However, a qualitative description of these characteristics provides a deeper understanding of the composition of the sample and highlights key patterns and trends. The majority of the respondents were male, with 18 out of the 20 key informants being men. A notable proportion of the participants (7 out of 20) had between 21 and 30 years of experience in their respective religious roles. The sample comprised a diverse range of religious denominations, including Catholic, Anglican, and Pentecostal traditions. Additionally, a substantial portion of the participants (7 out of 20) identified themselves as pastors.

### Religious denominations: A sub group within a religion that operates under a common name, tradition, and identity. Religion: a set of organized beliefs, practices, and systems that most often relate to the belief and worship of a supernatural being.

Table 3 shows the 2 major themes that emerged from the data: (a) Religious leaders’ knowledge regarding HIV prevention, and (b) Perceptions of religious leaders about HIV among young people.

### Theme 1: Religious leaders’ knowledge regarding HIV prevention

We investigated the religious leader’s understanding of HIV prevention and preventative methods, as well as their understanding of the modes of transmission of HIV.

### Understanding of HIV prevention

The participants expressed varying understandings of different aspects of how HIV is prevented. Below are voice excerpts from some of the study participants:

“I think this is taking care of young ones so that they won’t be exposed to HIV. “Male, R1.

“HIV prevention I would say in my local understanding as measures taken to see to it that the young people are protected from contracting the disease.” Female, R4.

“Of course, the prevention is abstinence and that is the only way how you can prevent it.” Male, R1.

Other participants, however, identified HIV prevention strategies as awareness creation, abstinence, faithfulness in marriage, and HIV testing before sex. This was mainly expressed by the participants, as seen in the below quote;

“You should first test your blood. Then you use a condom and you also have to be faithful to each other.” Female, R8.

Although some participants knew condom use as a way of HIV prevention, they did not recommend it for use among the youth. A participant said:

“I always see from other places, they encourage youth to use condoms but for us as the church, we don’t allow the use of condoms because we would be promoting sexual practices which is against our faith and it is not proper at all.” Male, R7.

### Understanding of modes of transmission of HIV

Knowledge regarding HIV was mostly expressed in terms of modes of HIV transmission. That is, participants were able to give a comprehensive account of how HIV is spread from one person to another. They noted that the greatest number of people get HIV through unprotected sexual intercourse as a route for HIV transmission. Some of the participants said that…

“…HIV spreads through the sexual relationships between girls and boys. For if a girl falls in love with a boy and has unprotected sexual intercourse, the infected person can transmit HIV to the other.” Male, R7.

Some religious leaders felt they lacked knowledge of HIV prevention methods, which limited their involvement in efforts towards HIV response among young people. A participant said that:

“Most of us lack knowledge on HIV prevention. We should be taught various HIV prevention measures to update our knowledge of HIV. It will make us understand and help in HIV prevention. Male, R18.

The same view was also shared by another key informant who reported that;

“We need to have more knowledge about this. A leader has to have general knowledge, to respond to youth questions. We have to study enough ….” Male, R10.

### Theme 2: Perceptions of religious leaders about HIV among young people.

The perceptions of religious leaders about HIV risk acquisition among youngsters and prevention measures were investigated. The participants were questioned regarding their perceptions and concerns regarding HIV prevention among young people.

### Religious leaders’ HIV risk perception among young people

Some study participants believed that young adults as well as adolescents were at a greater risk of acquiring HIV. The high risk was associated with early sexual debut, fornication, and inadequate knowledge of HIV prevention. One of the study participants narrated that:

“The danger of HIV among girls and boys starts from the age of 12 years. Secondly, they are entering into fornication when they are young, and they copy bad dress codes.” Male, R6.

In addition, another participant also said;

“The youth are at a very high risk of contracting HIV. And they are not only contracting HIV but are also spreading it.” Male, R9.

### Perceived roles of religious leaders in HIV prevention

In the interviews, when asked about how the religious leaders perceived their roles in preventing HIV among young people, a predominant view of their responsibilities included mentoring the youth, setting an example, speaking out against HIV, raising awareness, and working with medical professionals. One of the participants said:

“Our role is to teach people in God’s way, teaching them using the bible while using the word of God against sin… so we teach widely about sin, how we can prevent sin such as fornication which leads to HIV infection. So, we teach, sensitize, and, normally encourage them.” Male, R10.

Another participant also said:

“Religious leaders are the first key people to create awareness of HIV positive to the young and they are the most important people and they play an important part to the communities because they have access to teach the youth. Male, R3.

Some participants perceived that HIV prevention starts with religious leaders being exemplary. A number reported that by being exemplary, young people could be inspired and empowered to prevent the spread of HIV. A participant said:

“In my view, ……. to live an exemplary life to the young people and it’s my role to mentor them and empower them to be more careful with HIV.” Male, R13.

Some people perceived that breaking the silence surrounding HIV and openly discussing it in public was essential to preventing it among young people. One participant narrated:

“As a religious leader, I believe that the only way to prevent HIV is to inform young people about HIV honestly and openly to expand on their knowledge of the disease. Male, R20.

A few of the participants reported that one of the most important aspects of HIV prevention is having medical experts speak to the youth. Some even went so far as to say that they occasionally invite medical professionals to speak with the young people and inform them about the risks and modes of HIV infection. One participant said:

“What we always do in the church program, there are weeks that sometimes we dedicate to the medics to come and handle health-related issues for the whole church…. we let them come and talk about the health issues, so if the issue is about HIV, they will talk about it.” Male, R19.

Concerns about HIV prevention among young people

Participants expressed various concerns and held beliefs about HIV prevention among young people. Thus, they expressed views such as the fear of God. A good number of respondents reported that if the young people feared God, there would be fewer cases of HIV infection among them. Additionally, they stated that adhering to religious principles and practices could help lower the frequency of new HIV infections among the youth. One participant narrated that:

“If they are aware of the fear of God, they will turn away from all the issues ………. If these young people adopt all of these preventive measures, they will, in my opinion, live longer, be healthier, and fulfill all the plans and purposes that God has for them.” Male, R3.

The need to involve parents, school administrators, and other stakeholders in educating young people about HIV prevention was also mentioned by some of the participants. They expressed concern that young people are susceptible, vulnerable, and unreceptive to advice. Some of them said this:

“… In the prevention of HIV, there is a need to join hands among parents, among school leaders, among political leaders, teachers or government leaders and should talk about the prevention of HIV in public” Male, R5.

Although religious leaders make efforts to prevent HIV infections among the youth, the youth most often don’t cooperate with them. Some of the religious leaders perceived the young people as very rebellious. They further reported that young people do not listen to advice from family members. One of the key informants thus said:

“……they should stay away completely from sex because I have seen how the youth are behaving today, the way they talk, and not listen to the family yet they are very young. They cannot understand…. awareness should be given every day … so people should not keep quiet, we should not keep quiet.” Male, R1.

Another participant also reiterated that:

“We face difficulties. The first one is when we gather the youth and speak to them as we always do, but they choose not to listen.” Male, R11.

### Perceived enhancers for participation in HIV prevention

When asked how easy it was to reach young people, one group of study participants perceived accessibility as easy. They further said the ease of access to young people is beneficial when discussing HIV prevention strategies. One participant said:

“…. we have access to young people, and also when we conduct outreach ministry where we get to interact with very many people including the young persons …” Male, R20.

Another also said;

“What I think about that is religious leaders may be of advantage because every time they are speaking to people, praying for them regularly, and counseling them.” Male, R17.

A dominant view from the interview shows that religious leaders perceived that they commanded much respect from their societies, which has a positive impact on preventing HIV in young people. The respect accorded to faith leaders motivates them to lay tireless efforts and strategies aimed at reducing HIV spread among young people. One participant said:

“… they believe in us when we talk, and they take it to be serious because they know we are representatives of Christ. So, when we talk, it means it’s the voice of Christ thus they hear and obey…” Male, R19.

The participants also stated that the congregation has a high regard for religious leaders and is more likely to follow their counsel. A participant said:

“The advantage is that being spiritual leaders, people trust us and that many religious leaders live by example, and thus their audience believes in them. “Male, R13.

### Perceived barriers to participation in HIV prevention

Nevertheless, religious leaders reported that they face numerous challenges that have become bottlenecks to the success of the implementation of HIV preventive initiatives among youth. They stated some of the challenges that these religious leaders face during their participation in HIV prevention, such as lack of transport, lack of finance, and inadequate equipment. They stated as follows:

The study participants highlighted a challenge of transport, which limits the access of religious leaders to young people. One participant said:

“…….. for me to go down there, I need to have transport means, because these peoples’ homes are too far, too distant, and very many. That is the problem that I get, and sometimes I even think of just giving up.” Male, R5.

In addition, some of the religious leaders perceived the lack of financial support as a major hindrance in providing HIV prevention strategies to young people through activities like outreaches. A participant narrated:

“Another challenge is financial problems; we don’t have enough financial support.” Male, R2.

Another participant said:

“So, the problem is finance because these people are many which makes it hard to visit all the groups of the youth.” Male, R5.

On the other hand, a section of participants perceived the lack of equipment such as charts, and posters to limit their efforts on HIV prevention messages among young people. A participant narrated that:

“…. sometimes we are always limited with the facilities, the equipment, and the things that are required providing HIV prevention messages to the young people…” Male, R19.

Similarly, another participant said:

“I get the problem of not having the tools for work for example, we don’t have blackboards or even posters that we can use to help these people.” Male, R5.

Sometimes religious leaders are conflicted by religious beliefs and doctrines which are against some of the prevention measures against HIV, like condoms. A participant said:

“As I talked about the use of condoms, we cannot be advertising agents and leave it open for them like that to use it. We would be walking away from our principles though we don’t discourage.” R19

### Solutions to the challenges faced

During the discussion with the participants, ideas about how to help religious leaders overcome obstacles emerged, including different ways to empower them in their efforts to urge the youth to use HIV prevention strategies. It’s interesting that some of the solutions called for ongoing counseling, proximity to youth, and serving as a role model. Some leaders said that they always keep talking to and counseling young people. This would help increase young people’s awareness of HIV. One participant said:

“We are overcoming these challenges by frequently talking with these youths for example every Sunday like one or two words on counseling and guidance, and constantly preaching that HIV is there.” Male, R5.

Additionally, some religious leaders emphasized that getting close to the young people would help them understand them.

“Another thing is being close to those young ones, which puts me in a position to know their weaknesses.” Female, R4.

Apart from being close to the young, if religious leaders serve as a good example to the youth would enable them to learn and follow their spiritual leaders.

“I must set an example for others. When you want to act as an example, you should be serious and mean what you say because people will eventually follow you if they see you doing it.” Male, R1.

### Suggestions about how to strengthen HIV prevention

Fascinatingly, study participants highlighted how they perceived that HIV prevention can be strengthened among young people. Various views emerged about how to increase religious leaders’ participation in combating HIV spread, some of which included training the religious leaders, facilitating them in terms of transport, and giving them the necessary equipment.

Furthermore, one participant emphasized the fact that religious leaders need to be updated about HIV all the time. In their own words, they said:

“You might not be able to fill in the blanks, but you will need to have some knowledge. So, one thing I am doing diligently is learning more about HIV and AIDS.” Male, R9.

Another participant also said:

“…….. for me to go down there, it needs me to have transport means, because these peoples’ homes are too far, too distant, and very many.” Male, R5.

Some of the participants reported that greater milestones would be reached in HIV prevention among the youth if religious leaders worked together with healthcare workers. Some of the study participants suggested that they would prefer inviting healthcare providers to their various places of worship to train the religious leaders and also talk to the young people directly. One participant said:

“As religious leaders, we request that the medical personnel should spare some time and come and train us or help us talk to the young people.” Male, R11.

Last but not least, the majority of the respondents reported that supplying teaching materials to different places of worship and religious authorities would present a chance to provide young people with accurate information. Some study participants preferred receiving equipment to use when instructing and educating children. A participant said:

“They should give us work equipment, like posters having teaching pictures, pens, and books….” Female, R8.

## Discussion

Our study aimed to investigate the knowledge and perceptions held by religious leaders regarding HIV prevention among young people in Lira city and district. The findings indicated that participants generally possessed a relatively good understanding of HIV, its preventative measures, and modes of transmission. This knowledge level could be attributed to their involvement in HIV activities and exposure to information through various media channels (13, 24). Similar findings have been reported in studies conducted among faithbased organizations in Mozambique and Nigeria, suggesting a common trend across different regions (13, 24).. However, it’s essential to note that while religious leaders had a good understanding of certain aspects of HIV prevention, their knowledge was not comprehensive. Their knowledge was limited to religious beliefs.

Participants in our study demonstrated familiarity with behavioural prevention measures such as abstinence, faithfulness in marriage, and condom use as HIV prevention methods. However, most were hesitant to endorse condom use among youth, preferring to emphasize abstinence and faithfulness. This suggests a tendency among religious leaders to prioritize certain prevention methods over others, potentially neglecting the effectiveness of comprehensive approaches that include condoms, male circumcision, pre-exposure prophylaxis (PrEP), post-exposure prophylaxis (PEP), and other strategies. Thus, it is imperative for awareness campaigns led by religious institutions to promote a diverse range of HIV prevention strategies, rather than exclusively advocating for abstinence and faithfulness, which have shown limited efficacy in curbing HIV transmission.

Despite their awareness of various HIV prevention methods, participants in our study held some misconceptions regarding certain prevention strategies, echoing findings from previous research conducted in Nigeria (13). These misconceptions underscore existing knowledge gaps among religious leaders that may impede the success of HIV preventive initiatives and programs. Therefore, regular workshops and timely training on HIV should be provided to religious leaders to enhance their understanding of HIV prevention strategies. This would enable them to disseminate accurate information about HIV prevention and promote behavior change among young people effectively.

The majority of religious leaders in our study perceived themselves to have a significant impact on combating youth-related HIV spread. Their proximity to local communities, along with the respect and status they hold in society, affords them a significant opportunity to contribute to HIV prevention efforts (25). This role encompasses teaching morals, raising awareness about HIV, addressing the silence surrounding the topic, and collaborating with medical personnel to implement prevention programs targeting young people. Previous studies have highlighted the effectiveness of teaching and preaching about prevention strategies by religious leaders and their impact on HIV reduction programs (25, 26). Such motivation stems from the recognition that young people are at risk of HIV due to factors such as early sexual debut and unprotected sex. Therefore, religious leaders can leverage their influence to impart moral values and encourage safer sexual behaviors among young people.

However, our findings also revealed several barriers to the active participation of religious leaders in implementing HIV preventive initiatives among young people. These include conflicting religious beliefs, inadequate knowledge about HIV prevention, and insufficient training on HIV prevention methods (7). Religious beliefs significantly influence the decisions of religious leaders when recommending certain HIV prevention strategies, with some refraining from promoting condom use due to religious convictions (27, 28). This implies that congregations may receive limited information regarding alternative HIV prevention strategies, potentially hindering comprehensive prevention efforts. Addressing these barriers requires ongoing training and support for religious leaders, as well as the integration of HIV prevention initiatives into religious institutions’ agendas.

Despite the willingness of religious leaders to collaborate with healthcare providers to raise awareness about HIV prevention, significant challenges remain unaddressed. Financial constraints, inadequate resources, and logistical challenges hinder the effective participation of religious leaders in HIV prevention efforts (24). Efforts to address these challenges must involve multisectoral collaboration, with religious leaders being key players in the national HIV response (29). By providing religious leaders with ongoing training, resources, and support, they can effectively relay accurate information about HIV prevention to their communities, ultimately contributing to the reduction of HIV transmission among young people.

## Conclusion and recommendations

In conclusion, religious leaders’ knowledge of HIV prevention is limited by their religious beliefs. However, they are more knowledgeable about HIV prevention by way of no sex before marriage, abstinence, and faithfulness to a married partner. Additionally, they perceive a challenging role and think HIV among young people can be appropriately prevented by behavior change.

While religious leaders play a crucial role in raising awareness and promoting behavior change, addressing existing knowledge gaps and barriers to their participation is essential for the success of HIV prevention initiatives. Multisectoral collaboration, ongoing training, and support for religious leaders are vital for strengthening their contributions to HIV prevention efforts and achieving national HIV targets. In addition, it is also vital that future religious’ HIV prevention awareness initiatives emphasize not only behavioral measures but also condom use, PEP, and PrEP. However, it’s important to acknowledge the limitations of our study, including the small study setting and potential biases, which may impact the generalizability of our findings to other contexts. In addition, it is important to explore the alternative perspectives of young people’s experiences and perspectives regarding religious leaders’ roles in HIV prevention.

## Figures and Tables

**Table 1.** Data collection tool

S/N	Interview question	Probe
1.	I would like to know your age, religious affiliation, sex, position you hold and your years of experience in service.	
2.	I would like to know how you understand by HIV prevention among young people.	Understanding of HIV prevention. Measures for HIV prevention.
3	I would like to know how you understand HIV transmission among young people.	Modes of HIV transmission
4	As a religious leader, I would like to know how you perceive the HIV risk among the young people.	
5	Please discuss what religious leaders think regarding their roles in HIV prevention among young people. I would like to know your own views regarding the roles of religious leaders in HIV prevention among young people.	
6	Please discuss any concern, beliefs you have towards HIV prevention among young people.	How easy or hard is it to implement HIV prevention services among young people.
7	Let’s discuss the advantages you think religious leaders have over secular organization as far as HIV/AIDS prevention among young people is concern.	
8	Let’s talk about any challenges you face in extending those services to young people and how you overcome them.	
9	As a religious leader, what things can you do to strengthen your role in HIV/AIDS prevention interventions?	

Interview guide regarding religious leaders’ attitudes and knowledge of HIV prevention among youth in Northern UgandanLira.

**Table 1.** Socio-demographic characteristics of the participants (n=20).

Variable	Category	Frequency(n=20)

**Religious denomination**	Catholic	5
	Anglican.	5
	Moslem	2
	Seventh Day Adventist	3
	Pentecostal.	5
**Religious position**	Reverend.	2
	Catechist.	5
	Ordinand	2
	Pastor	7
	Priest	2
	Sheikh	1
	Imam	1
**Sex**	Female	2
	Male	18
**Years of experience**	<10 years	6
	10–20 years	6
	21–30 years	7
	Above 30 years	1

Sociodemographic characteristics of the participants.

**Table 3:** Summary of themes and subthemes

Theme	Sub-theme	category
Religious leaders’ knowledge regarding HIV prevention	a) Understanding of HIV prevention b) Understanding of modes of transmission of HIV	
Perceptions of religious leaders about HIV among young people.	Religious leaders’ HIV risk perception among young people	
	Perceived roles of religious leaders in HIV prevention	mentoring the youth, setting an example, speaking out against HIV, raising awareness, and working with medical professionals.
	Concerns about HIV prevention among young people	
	Perceived enhancers for participation in HIV prevention	Ease of access to young people, the respect accorded to faith leaders, being trusted by the congregation
	Perceived barriers to participation in HIV prevention	Lack of transport, lack of finance, and inadequate equipment
	Solutions to the challenges faced	Continuous counseling, proximity to youth, and serving as a role model
	Suggestions about how to strengthen HIV prevention	Training the religious leaders, facilitating them in terms of transport, and giving them the necessary equipment

A summary of the themes, sub-themes, and categories of knowledge and perceptions of religious leaders towards HIVprevention among young people in Lira district.

## Data Availability

The corresponding author will provide all the research materials utilized in this work upon request.
